# Generation, analysis and functional annotation of expressed sequence tags from the sheepshead minnow (*Cyprinodon variegatus*)

**DOI:** 10.1186/1471-2164-11-S2-S4

**Published:** 2010-11-02

**Authors:** Mehdi Pirooznia, Alexander Pozhitkov, Edward J Perkins, Youping Deng, Marius Brouwer

**Affiliations:** 1 School of Medicine, Johns Hopkins University, Baltimore, MD, 21287, USA; 2 Department of Coastal Sciences, University of Southern Mississippi., Hattiesburg, MS, USA; 3 Environmental Laboratory, U.S. Army Engineer Research and Development Center, Vicksburg, MS, 39180, USA; 4 SpecPro Inc., 3909 Halls Ferry Rd, Vicksburg, MS, 39180, USA

## Abstract

**Background:**

Sheepshead minnow (*Cyprinodon variegatus*) are small fish capable of withstanding exposure to very low levels of dissolved oxygen, as well as extreme temperatures and salinities. It is an important model in understanding the impacts and biological response to hypoxia and co-occurring compounding stressors such as polycyclic aromatic hydrocarbons, endocrine disrupting chemicals, metals and herbicides. Here, we initiated a project to sequence and analyze over 10,000 ESTs generated from the Sheepshead minnow (*Cyprinodon variegatus*) as a resource for investigating stressor responses.

**Results:**

We sequenced 10,858 EST clones using a normalized cDNA library made from larval, embryonic and adult suppression subtractive hybridization-PCR (SSH) libraries. Post- sequencing processing led to 8,099 high quality sequences. Clustering analysis of these ESTs indentified 4,223 unique sequences containing 1,053 contigs and 3,170 singletons. BLASTX searches produced 1,394 significant (E-value < 10^-5^) hits and further Gene Ontology (GO) analysis annotated 388 of these genes. All the EST sequences were deposited by Expressed Sequence Tags database (dbEST) in GenBank (GenBank: GE329585 to GE337683). Gene discovery and annotations are presented and discussed. This set of ESTs represents a significant proportion of the Sheepshead minnow (*Cyprinodon variegatus*) transcriptome, and provides a material basis for the development of microarrays useful for further gene expression studies in association with stressors such as hypoxia, cadmium, chromium and pyrene.

## Background

Sheepshead minnow (*Cyprinodon variegatus*) are small teleost fish found in western Atlantic estuaries extending from Cape Cod, MA southward to Mexico and parts of the Caribbean. Under experimental conditions, *C. variegatus* are capable of withstanding exposure to very low levels of dissolved oxygen (DO) (< 1 mg/l, ref. [1]), as well as extreme temperatures (0.6 to 45°C, refs. [2], [3]) and salinities (0 to 142 ‰, ref. [4]). Sheepshead minnow is an estuarine fish model to investigate genomic and proteomic responses to hypoxia and co-occurring compounding stressors such as polycyclic aromatic hydrocarbons, endocrine disrupting chemicals, metals and herbicides. Current efforts to understand mechanisms behind Sheepshead minnow’s tolerance to these conditions are hampered by a lack of genetic data available for development of transcriptome analysis tools.

Expressed sequence tag (EST) analysis is one of the most effective means for gene discoveries, gene expression profiling, and functional genome studies [5]. The objectives of this study were to create cDNA libraries suitable for the analysis of ESTs and to generate an EST resource for Sheepshead minnow to allow cDNA-based design of microarrays in order to provide genomic resources for the analysis of differentially expressed genes. This EST resource should provide the material basis for the development of microarrays and serve as a platform for its functional genomics studies. Here, we describe a collection of more than 10,000 ESTs generated from the Sheepshead minnow, putatively representing 4,223 different transcripts after sequence assembly. To facilitate gene identification and functional genomics studies, the EST set has been annotated using the structured vocabulary provided by the Gene Ontology Consortium (2001), based on molecular studies of gene function and metabolic pathway analysis based on KEGG (Kyoto Encyclopedia of Genes and Genomes) pathway database.

## Results

### cDNA library and EST sequence analysis

**Figure 1:**
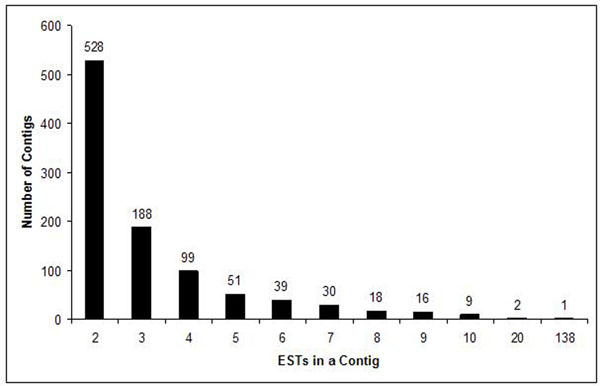
Distribution of 4929 ESTs in 1053 contigs

A total number of 10,858 ESTs were sequenced. After cleansing, 75% were retained (8,099). The observed rejection rate (25%) is typical for high throughput sequencing projects [6]. The number of ESTs in assembled fragments (contigs) was 45% of the total (4,929), which was assembled into 1,053 contigs. Singlets consisted 30% of the total (3,170). All non-cleansed sequences were submitted to dbEST database at NCBI under the accession numbers GE329585 to GE337683. The distribution of the ESTs in contigs is shown in Figure 1. Singlets and contigs together constituted 4,223 unique sequences. Among the most represented genes in the cDNA libraries were parvalbumin, glycosyl transferase, transferrin, myosin, and vitellogenin (Table 1).

**Table 1:** The most represented genes in the cDNA library

ESTs	GI	E-value	Organism	Description
138	71897458	4.00E-47	Oreochromis mossambicus	Parvalbumin
109	153009546	4.00E-93	Ochrobactrum anthropi ATCC 49188	Glycosyl transferase family 2
102	153009546	2.00E-80	Ochrobactrum anthropi ATCC 49188	Glycosyl transferase family 2
71	157694802	1.00E-41	Lates calcarifer	Apolipoprotein
48	50953783	2.00E-40	Rivulus marmoratus	Parvalbumin 2
44	89475215	0	Oreochromis niloticus	Transferrin
38	60685065	4.00E-80	Oryzias latipes	Myosin light chain 2
34	22002418	3.00E-66	Oryzias latipes	Embryonic beta-type globin
34	21694043	1.00E-106	Oreochromis mossambicus	Muscle-type creatine kinase CKM2
32	62241082	1.00E-166	Gambusia affinis	Vitellogenin
31	157065018	2.00E-67	Spondyliosoma cantharus	Elastase-like serine protease
30	127461921	0	Cyprinodon rubrofluviatilis	Cytochrome oxidase subunit I

**Table 2:** Homology analysis of the unique sequences based on BLASTX against nr database of NCBI.

E-value	ESTs	%
<10-100	30	1
10-100 < E ≤ 10-50	280	7
10-50 < E ≤ 10-20	644	15
10-20 < E ≤ 10-5	750	18

Total significant	1394	33
Total non-significant	950	22
No hits	1879	44
Total ESTs	4223	

### Comparative sequence analysis

The unique sequences (4,223) were used to search the Non-redundant protein database at the National Center for Biotechnology Information (NCBI). Eight percent of the total 4,223 unique sequences matched the nr database with a cut-off e-value of less than 10^-50^, 15% matched at the e-value between 10^-50^ and 10^-20^, and, 18% between 10^-20^ and 10^-5^ (Table 2) had significant BLASTX hits. For a total of 950 (22%) no significant hits were found. And finally, the remaining 1,879 sequences had no match with the nr database at all. Table 3 shows the distribution of the significant BLASTX hits of the unique sequences per organism. The findings suggest that there are many common DNA fragments and genes among different species of fish.

**Table 3:** Distribution of significant homologous matches (e ≤ 10^-5^) of the unique sequences per organism.

Organism	Hits
Tetraodon nigroviridis	455
Danio rerio	277
Oryzias latipes	30
Takifugu rubripes	29
Fundulus heteroclitus	29
Sparus aurata	28
Ochrobactrum anthropi	25
Pagrus major	24
Paralichthys olivaceus	16
Solea senegalensis	15
Cyprinodon rubrofluviatilis	14
Xenopus laevis	14
Oncorhynchus mykiss	13
Equus caballus	11
Oreochromis mossambicus	11
Ictalurus punctatus	10

**Table 4:** Number of genes involved into reconstruction of KEGG pathway maps (partial list, number of ESTs >4).

Pathway	Pathway Name	ESTs
00190	Oxidative phosphorylation	81
03010	Ribosome	53
01430	Cell junctions	21
04530	Tight junction	11
04520	Adherens junction	11
04510	Focal adhesion	11
04670	Leukocyte transendothelial migration	11
00280	Valine, leucine and isoleucine degradation	9
00071	Fatty acid metabolism	9
00632	Benzoate degradation via CoA ligation	7
00350	Tyrosine metabolism	6
00360	Phenylalanine metabolism	6
00120	Bile acid biosynthesis	6
00062	Fatty acid elongation in mitochondria	6
00330	Arginine and proline metabolism	5
04020	Calcium signaling pathway	5

### Functional classification

**Figure 2:**
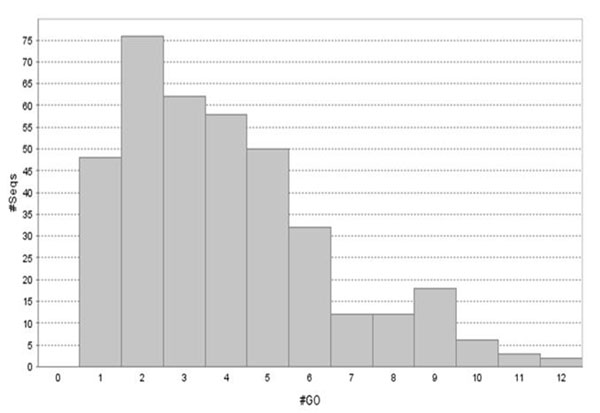
Distribution of the number of annotated sequences versus the number of GO terms

**Figure 3:**
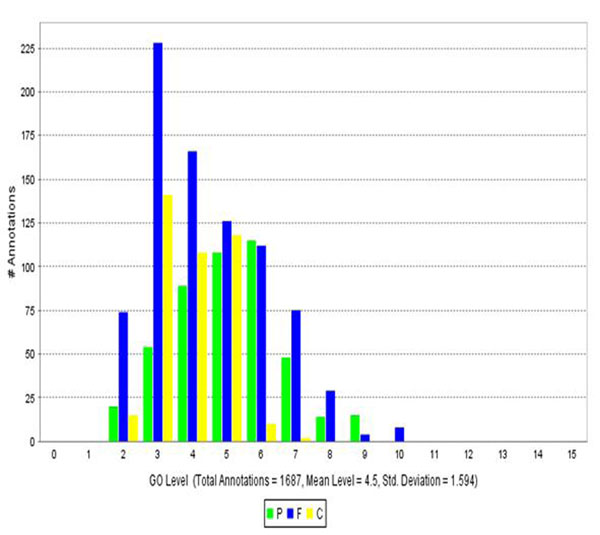
The GO-level distribution of the annotated sequences, P - biological process, F - molecular function, C - cellular component.

**Figure 4:**
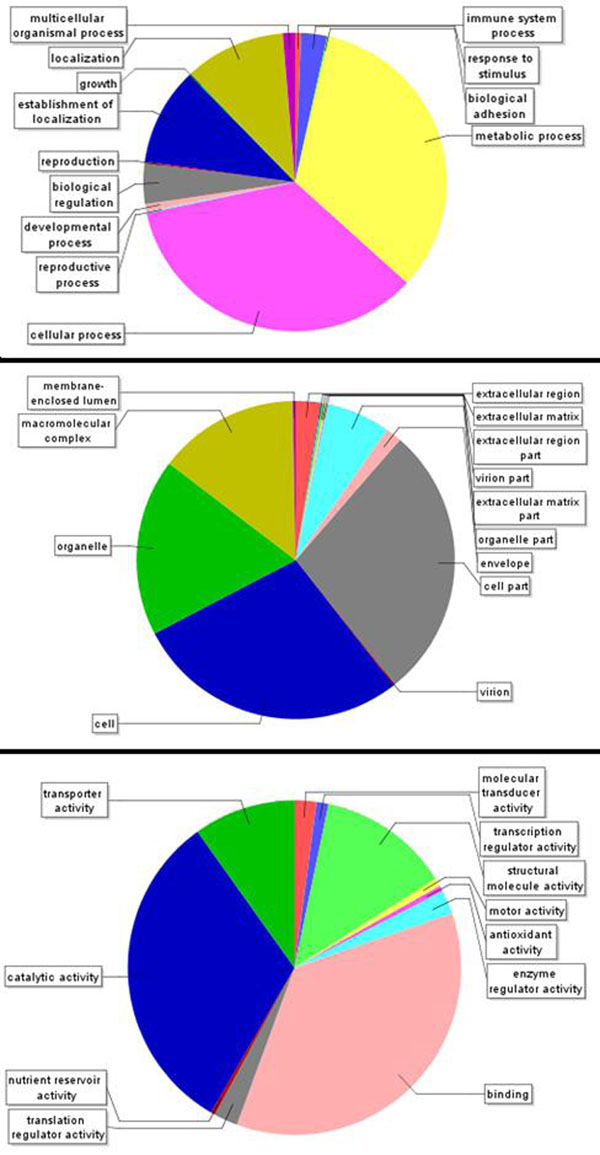
Pie charts of the second-level GO terms. Each of the three GO categories is presented including (left to right): biological process, cellular component and molecular function

Gene ontology (GO) [7] annotation was employed to interpret the function of the sequences cloned from *Cyprinodon variegatus.* Several sequences had as many as 12 GO terms associated with them (Figure 2). As expected, the highest number of annotations come from the molecular function category (i.e. function of a gene product) (Figure 3), We determined the percentages of second level GO terms among three major categories: biological process, cellular component and molecular function (Figure 4). Among the biological process GO terms, 33% and 34% were related to cellular and metabolic processes respectively. In the category of molecular function, the vast majority were involved into binding (36%) and catalytic activities (31%). Under the category of cellular components, 64% of all GO terms corresponded to cell parts and organelle. Metabolic pathways were obtained from the KEGG (Kyoto Encyclopedia of Genes and Genomes) pathway database. Several KEGG pathways were represented by 5 or more ESTs (Table 4). The highest number of KEGG mappings was extracted from Oxidative phosphorylation and Ribosome pathways with 81 and 53 sequences respectively. In terms of Enzyme Commission number (EC number), our annotation contained 52 records corresponding to 31 unique EC numbers. The entire database is available in Supplement.

### Conclusions

We have produced 8,099 high quality Sheepshead minnow (*Cyprinodon variegatus*) EST sequences from multiple developmental stages (see methods). Sequence analysis indicated the presence of 4,223 unique sequences in the EST set. This should represent a significant fraction of the Sheepshead minnow genes, although the exact gene number of the fish is unknown at present. The majority of the unique EST sequences had similarities to known genes, especially Zebrafish, making them more amenable to functional analysis. The EST sequences should enhance the effectiveness of molecular studies, especially for gene expression profiling and design of microarrays. Additionally, the cluster and redundancy information should be useful for further subtraction of the most abundant transcripts included in the cDNA library, making further EST analysis more effective.

## Materials and methods

### Source of animals

Fish exposure protocols received prior approval from the University of Southern Mississippi Institutional Animal Care and Use Committee (#121 R02) and attempts to adhere to the Guidelines for the Use of Fishes in Research proposed by the American Fisheries Society were made whenever possible. Sheepshead minnows (*Cyprinodon variegatus*) used in the current study were either derived from an outbred, laboratory- reared stock maintained at the Gulf Coast Research Laboratory (GCRL), University of Southern Mississippi (Ocean Springs, MS) or were collected from estuarine waters located near Point Aux Chenes Road in Ocean Springs, Mississippi and acclimated to dilution/culture for at least one week. Prior to exposure, fish were maintained at 27°C in 15 ‰ salinity artificial seawater.

### Exposures for trimmer normalization libraries

Fertilized eggs were collected from adult, wild caught Sheepshead minnows (Cyprinodon variegatus), allowed to hatch in 15 ‰ salinity artificial seawater, transferred to Carolina bowls, and maintained for up to 7 days post hatch (dph). Larvae were removed from the Carolina bowls at 1, 3, 5, and 7 dph, preserved in RNA Later (Ambion, Austin, TX), and stored at -20°C until total RNA was extracted. Adult wild caught Sheepshead minnows female fish were injected with 50μL of chorionic gonadotropin followed by a second injection on the next day after the initial injection. The fish were maintained in normoxic conditions until the third day following the initial injection at which time the eggs were stripped from the females. The eggs were collected in a 50mL beaker containing just enough seawater to cover the eggs and fertilized using the dissected testes from male Sheepshead minnows. Viable embryos were maintained in 15 ‰ salinity artificial seawater, removed at 12, 24, 48, 72, and 96 hours post fertilization, homogenized in 750ul RNA STAT-60 (Tel-Test, Inc., Friendswood, TX), and stored at - 20°C until total RNA was extracted.

### Exposures for larval SSH libraries

Fertilized eggs were collected from the adult, laboratory reared stock of Sheepshead minnows and were allowed to hatch in 15 ‰ salinity artificial seawater. Egg collections were monitored daily and larval fish were collected within 6 hours of hatching and transferred to their respective exposure treatment. Thirty-five viable larvae were placed in retention chambers (15cm diameter petri dishes with a 400 micrometer nylon mesh collar) and exposed to the experimental or the control treatment over a period of 7 days. Experimental treatments of the larval fish included 9.5mg/L Cr(VI), 0.3mg/L Cd(II), 40μg/L pyrene, chronic 1.5mg/L DO, or cyclic 1.5mg/L DO using an intermittent flow-through system. Control treatments of the larval fish included saltwater control (chromium and cadmium), DMSO (pyrene), and 6-8mg/L normoxia (chronic and cyclic DO). Exposure temperatures were maintained at 26-28°C under a 16 h light:8 h dark photoperiod. Larvae were sampled at 1, 3, 5, and 7 days post hatch and stored in RNA Later (Ambion, Austin, TX) at -80°C until total RNA was extracted.

#### Exposures for Embryonic SSH libraries

Adult female fish were injected with 50μL of chorionic gonadotropin followed by a second injection on the next day after the initial injection. The fish were maintained in normoxic conditions until the third day following the initial injection at which time the eggs were stripped from the females. The eggs were collected in a 50mL beaker containing just enough seawater to cover the eggs and fertilized using the dissected testes from male Sheepshead minnows. After one hour, eggs were examined for fertilization and 25 viable, fertilized embryos were placed in retention chambers (15cm diameter Petri dishes with a 400 micrometer nylon mesh collar) and exposed to the experimental or the control treatment over a period of 4 days. Experimental treatments of the embryonic fish included 40μg/L pyrene, chronic 1.5mg/L DO, or cyclic 1.5mg/L DO using an intermittent flow-through system. Control treatments of the embryonic fish included DMSO (pyrene) and 6-8mg/L normoxia (chronic and cyclic DO). Exposure temperatures were maintained at 26-28°C under a 16 h light:8 h dark photoperiod. Embryos were sampled at 24, 48, 72, and 96 hours post fertilization and homogenized immediately in 750μl RNA STAT-60 (Tel-Test, Inc., Friendswood, TX) and stored at -80°C until total RNA was extracted.

#### Exposures for Adult SSH libraries

Adult fish (6-8 months old) were subjected to either hypoxia (1.5mg O2 /L) or normoxia (~8mg O2 /L) in 38L glass tanks using a semi- flow-through exposure system and a 16 h light:8 h dark photoperiod. Throughout the experiments, water temperature and salinity were maintained at 27± 1 °C and 15 ‰, respectively. To create the desired dissolved oxygen (DO) levels, nitrogen gas was used to deplete oxygen. Gas input was regulated by solenoids controlled by AquaController II units (Neptune Systems, San Jose, CA) receiving data from temperature and DO probes. Measurements of tank pH, temperature, salinity, and dissolved oxygen were recorded. Fish were separated into 8 tanks per exposure group (1.5mg DO/L hypoxia and ~8mg DO/L normoxia) at a density of 10 fish /tank. At 10 and 96 h, fish from 4 tanks per exposure group were removed, sacrificed, and dissected. Excised hepatic tissue was placed in RNA Later (Ambion, Austin, TX) and stored at -80°C until total RNA was extracted.

### Molecular biology procedures

#### RNA extraction

For isolation of total RNA, larval, embryonic, or tissue samples were homogenized in 750μl of RNA STAT-60 (Tel-Test, Inc., Friendswood, TX). Total RNA was extracted by two rounds of reagent treatment according to the manufacturer’s protocol. Following the second extraction, RNA was precipitated overnight in 100% isopropanol, pelleted at 12,000 x g and 4°C for 1 h, and washed twice in 70% ethanol. The RNA pellets were resuspended in RNA Storage Solution (Ambion, Austin, TX) and subjected to TURBO DNasefree (Ambion, Austin, TX) treatment to remove any contaminating DNA. The purity and quantity of the resulting total RNA was determined using the ND-1000 spectrophotometer (NanoDrop Technologies, Wilmington, DE). All total RNA samples used in this study possessed a 260/280nm ratio of greater than 1.8. The quality of the total RNA samples (ie. 28S/18S ratio, DNA contamination, and RNA degradation) was determined using the RNA 6000 Nano Chip Kit and the Agilent 2100 Bioanalyzer (Agilent Technologies, Palo Alto, CA). Total RNA samples were pooled and ethanol precipitated in preparation for poly A + mRNA extraction and stored at - 80°C. Poly A + mRNA was extracted from pooled total RNA samples using the Oligotex mRNA Kits (Qiagen, Valencia, CA). Total RNA was subjected to two rounds of mRNA purification according to the manufacturer’s protocol. The quality of the mRNA purification (ie. removal of rRNAs) was determined using the RNA 6000 Nano Chip Kit and the Agilent 2100 Bioanalyzer (Agilent Technologies, Palo Alto, CA). The purity and quantity of the resulting mRNA was determined using the ND- 1000 spectrophotometer (NanoDrop Technologies, Wilmington, DE). mRNA samples were pooled and ethanol precipitated in preparation for cDNA normalization, suppression subtractive hybridization, or cDNA library preparation and stored at -80°C.

#### Trimmer normalization libraries

Larval mRNA (0.5μg; 125ng/time point) and embryonic total RNA (1.0μg; 200ng/time point) were used to generate double stranded cDNA using the BD SMART™ PCR cDNA Synthesis Kit (Clontech, Mountain View, CA) following the manufacturer’s protocol for “SMART cDNA Synthesis for Library Construction”. Double stranded cDNA products were purified using the QIAquick PCR Purification Kit (QIAGEN, Valencia, CA) and 1300ng purified cDNA product was aliquotted and ethanol precipitated. SMART prepared double stranded cDNA was normalized using the Trimmer cDNA Normalization Kit (Evrogen, Moscow, Russia) by treatment with duplex-specific nuclease (DSN). Non-normalized cDNA samples were also prepared in the absence of DSN. Normalized and non-normalized cDNA products were amplified using the Advantage 2 PCR Kit (Clontech, Mountain View, CA) according to the Evrogen protocol. PCR products of the normalized and nonnormalized cDNA were stored at -20°C.

#### Suppression subtractive hybridization libraries

The PCR-Select cDNA Subtraction Kit (Clontech, Mountain View, CA) was used to prepare suppression subtractive hybridization (SSH) libraries of up- and down-regulated genes in larval, embryonic, and/or adult *Cyprinodon variegatus* following exposure to chromium, cadmium, pyrene, chronic hypoxia, or cyclic hypoxia. Two micrograms of poly A + was used to generate double stranded cDNA for all exposed and control treatments. Double stranded cDNA was restriction digested with Rsa I and used to generate both tester and driver cDNA for all samples. Forward and reverse subtractive hybridizations between exposed and control samples for each corresponding treatment were performed according to the manufacturer’s protocol and the final subtracted PCR products were stored at -20°C.

#### Cloning and plasmid DNA miniprep

PCR products generated from the Trimmer cDNA Normalization Kit (Evrogen, Moscow, Russia) and the PCR-Select cDNA Subtraction Kit (Clontech, Mountain View, CA) were cloned using the pGEM-T Easy Vector System (Promega, Madison, WI) and Electromax DH10B T1 Phage Resistant Cells (Invitrogen, Carlsbad, CA). Ampicillin resistant colonies were isolated by blue- white screening, cultured in 2X LB, and preserved by freezing at -80°C with glycerol. Plasmid DNA was extracted from selected colonies using the Montage Plasmid Miniprep96 Kit (Millipore) and following the manufacturer’s protocol. Plasmid DNA concentrations were determined using the ND-1000 spectrophotometer (NanoDrop Technologies, Wilmington, DE) and samples were stored at -20°C.

#### Sequencing

DNA sequences of the cloned PCR products were determined using the DTCS Quick Start Kit (Beckman Coulter, Fullerton, CA). One-half 20μl reactions were performed using 75ng of plasmid DNA template and a M13 (-40) forward primer according to the manufacturer’s protocol for DNA sequencing of plasmid DNA. Sequencing reactions were purified by ethanol precipitation and analyzed on a CEQ 8000 Genetic Analysis System using a 33-75B separation capillary array under the LFR-1 default settings.

### Bioinformatic analysis

#### EST processing

Sequencher (Gene Codes, Ann Arbor, Michigan, USA), Aligner (CodonCode, Dedham, MA, USA), and CAP (source) were utilized for sequence cleansing and assembly. Low quality sequences and vector sequences were be quickly removed from each EST clone by using these applications. The cleaned EST sequences were used to find overlap assembly of contiguous sequences. Phred [8] was used to perform base-calling. Phred read DNA trace data, called bases, assigned quality values to the bases, and wrote the base calls and quality values to output sequence files. Quality values for the bases were later used by the sequence assembly program, Phrap [8], to increase the accuracy of assembled sequences. We employed CodonCode Aligner (http://www.codoncode.com/aligner/) to remove low quality bases at both ends by setting quality score *QV ≥* 20 (or *Pe* ≤ 0.01). Vector and adaptor sequences were also trimmed because they can lead to incorrect assembly. We also used an in house implementation of VecScreen [11] to detect any partial vector contamination in our ESTs. Cap3 [12] was used to identify overlaps between sequences and assemble sequence fragments into a larger sequence [12]. Samples that can be joined together were assembled into contiguous sequences “contigs”. Four criteria were used to determine whether to accept or reject an alignment and overlap: (1) minimum percent identity ≥ 70%; (2) minimum overlap length ≥ 25 bps, (3) minimum alignment score; similar to previous but takes any mismatches into account, ≥ 20 bps; and (4) maximum gap size ≤ 15 bps. After assembly with Cap3, contigs with more than three ESTs were examined in Consed [13] to eliminate additional missassemblies not resolved by Cap3.

#### EST comparative analysis and functional assignment

We performed comparative analysis using NCBI blastx [14] with the unique sequences (including assembled contigs and singletons). A local implementation of BLAST server was used to search against the NCBI’s non-redundant peptide sequence database. We set up a cut off value and discarded hits with an *E*-value < 10-5. To assign putative functions to the unique sequences, we employed blast2go [15], [16], GOTM [17], GOfetcher [6] and GOstat [18] to extract the GO hierarchical terms of their homologous genes from the protein databases. Meanwhile, we also mapped the unique sequences to metabolic pathways in accordance with the KEGG [19]. Enzyme commission (EC) numbers [20] were obtained and used to putatively map unique sequences to specific biochemical pathways.

## Competing interests

The authors declare that they have no competing interests

## Authors’ contributions

AP and MB initiated the study. AP performed the exposure and conducted RNA isolation, cDNA cloning and sequencing. MP, AP and YD designed the framework for data analysis and interpretation of data. MP and YD designed and implemented cleansing and assembling process, blast extraction, gene ontology, and pathway analysis. MP implemented a local blast for EST data analysis and participated in cleansing and assembling process. MP and AP drafted the original manuscript. YD, and MB coordinated and directed the project. All authors have participated in the final editing and have read and approved the final manuscript.
